# A longitudinal cline characterizes the genetic structure of human populations in the Tibetan plateau

**DOI:** 10.1371/journal.pone.0175885

**Published:** 2017-04-27

**Authors:** Choongwon Jeong, Benjamin M. Peter, Buddha Basnyat, Maniraj Neupane, Cynthia M. Beall, Geoff Childs, Sienna R. Craig, John Novembre, Anna Di Rienzo

**Affiliations:** 1 Department of Human Genetics, University of Chicago, Chicago, IL, United States of America; 2 Oxford University Clinical Research Unit, Patan Hospital, Kathmandu, Nepal; 3 Mountain Medicine Society of Nepal, Kathmandu, Nepal; 4 Department of Anthropology, Case Western Reserve University, Cleveland, OH, United States of America; 5 Department of Anthropology, Washington University in St. Louis, St. Louis, MO, United States of America; 6 Department of Anthropology, Dartmouth College, Hanover, NH, United States of America; National Cheng Kung University, TAIWAN

## Abstract

Indigenous populations of the Tibetan plateau have attracted much attention for their good performance at extreme high altitude. Most genetic studies of Tibetan adaptations have used genetic variation data at the genome scale, while genetic inferences about their demography and population structure are largely based on uniparental markers. To provide genome-wide information on population structure, we analyzed new and published data of 338 individuals from indigenous populations across the plateau in conjunction with worldwide genetic variation data. We found a clear signal of genetic stratification across the east-west axis within Tibetan samples. Samples from more eastern locations tend to have higher genetic affinity with lowland East Asians, which can be explained by more gene flow from lowland East Asia onto the plateau. Our findings corroborate a previous report of admixture signals in Tibetans, which were based on a subset of the samples analyzed here, but add evidence for isolation by distance in a broader geospatial context.

## Introduction

**Fig 1:**
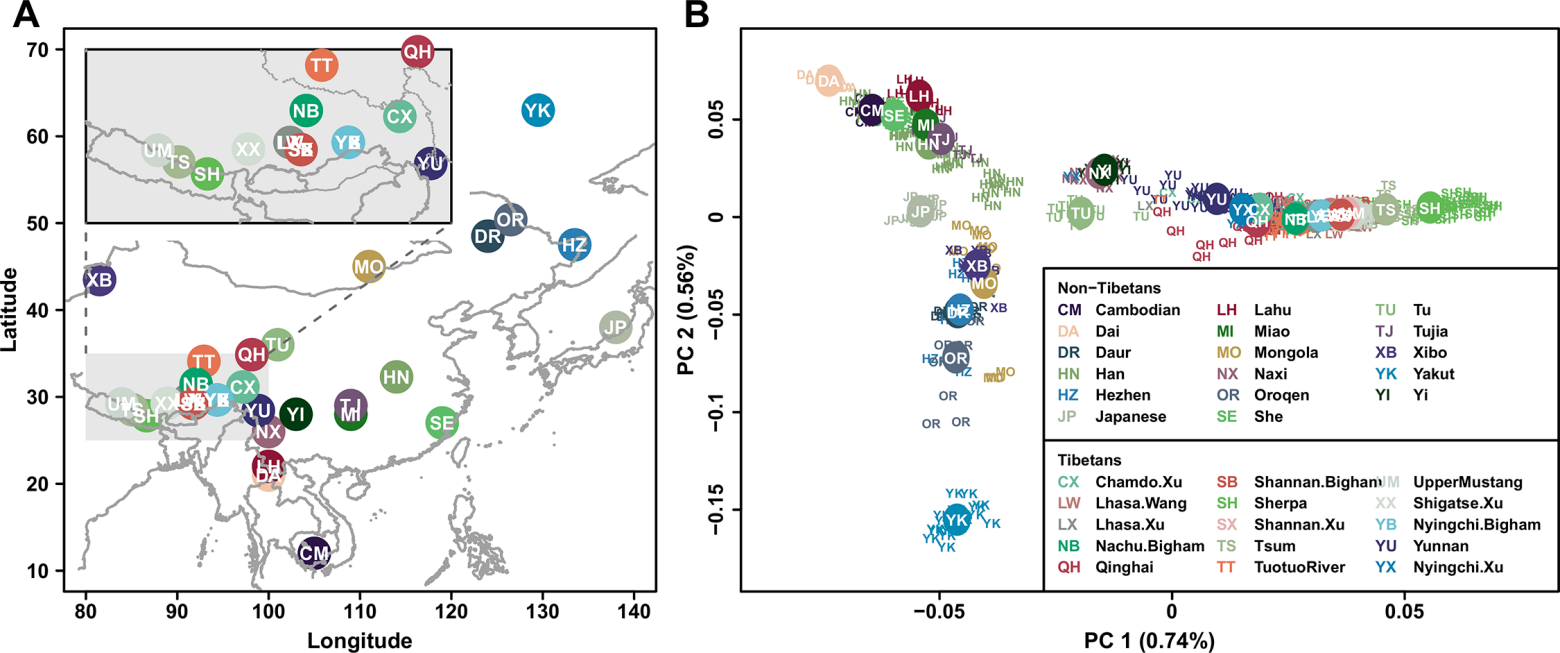
East Asian and Tibetan populations used in this study. (A) Geographic location of each populations are shown, with an inset for a zoom in of Tibetan samples. Dotted lines in the inset mark the borders of Chinese provinces. The map was generated using R package “mapdata” v2.2–6 [15]. (B) The first two PCs of East Asian and Tibetan genetic variation are shown. PC 1 separates Tibetans from the rest and PC 2 is consistent with a north-south cline across East Asia. Colored circles mark mean positions of populations. Numbers in parenthesis represent proportion of total variation explained by each PC.

The Tibetan plateau covers a vast geographic area stretching roughly 2,500 km in east-west direction and 1,000 km in north-south direction, corresponding to a quarter of the size of the United States. It is home to several million ethnic Tibetans as well as other ethnic groups. Recent genomic studies of ethnic Tibetans have focused on their adaptations to high-altitude hypoxia, and have discovered oxygen homeostasis genes harboring strong signatures of recent positive selection, such as *EGLN1* (egl-9 family hypoxia-inducible factor 1) and *EPAS1* (endothelial PAS domain protein 1) [1,2,3]. The genetic history of Tibetans also has attracted attention in population genetics, due to the presence of autochtonous lineages of uniparental markers with ancient coalescent times dating back to at least 22 kya, such as the Y haplogroup D [4,5,6,7]. Only a handful of studies used genome-wide data and they proposed markedly different demographic models, especially in terms of divergence time between Tibetans and Han Chinese [3,8]. While one study estimated Tibetan-Han divergence of 2,750 years ago using site frequency spectrum data from exome sequencing [3], the other suggested an initial split between the Sherpa and Han up to 20 kya [8] based on trajectories of effective population size inferred by the Pairwise Sequentially Markovian Coalescent model [9], and detected widespread admixture signals in multiple Tibetan samples [8]. The Sherpa, meaning “eastern people” in Tibetan, can be considered a subgroup of Tibetan populations for many reasons: they share aspects of Tibetan culture, religion and ways of life and speak a language that belongs to the Tibeto-Burman family. In addition, historical records suggest that at least part of the Sherpa population originated from a migration from eastern Tibet 4–6 centuries ago [10]. The results of a previous genetic study supported the idea that the Sherpa are closely related to the larger high-altitude Tibetan population [8]. Here, we investigated the genetic structure of Tibetan populations using genome-wide variation data of 338 Tibetan individuals, including Sherpa, from 12 localities in the plateau, spanning over 1,500 km east-west and 700 km north-south (Fig 1A, S1 Table) [1,2,8,11,12,13,14].

## Results and discussion

We generated novel genome-wide variation data for 53 Tibetans from highland Nepal and compiled these new data with published genome-wide variation data from 285 Tibetans (including Sherpa) and with world-wide population data from the Human Genome Diversity Panel [16] and the 1000 Genomes Project [17]. The final data set includes 69,427 SNPs and 3,780 individuals (Materials and Methods, S1 Table).

**Fig 2:**
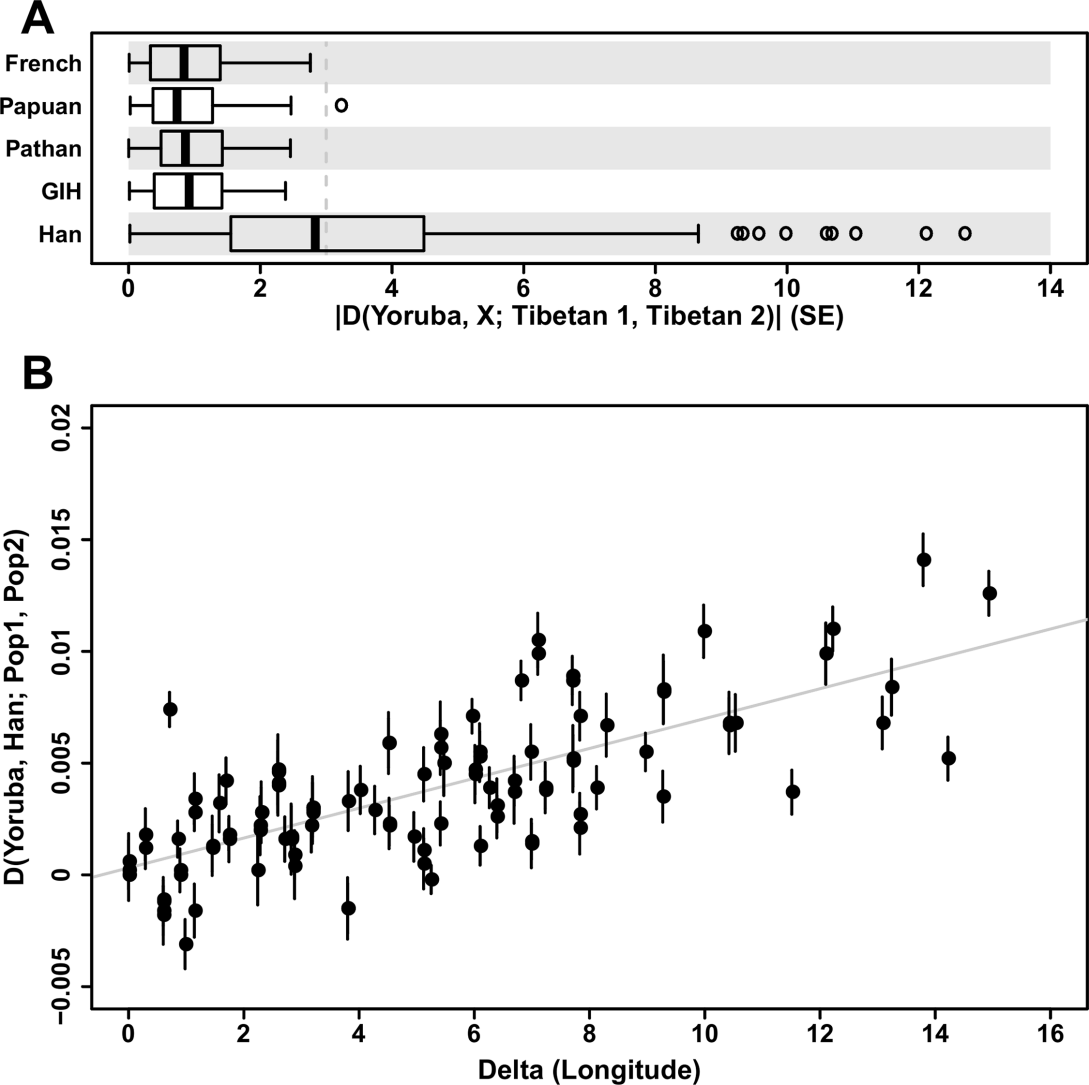
Patterson’s D statistics applied to pairs of Tibetan samples, in the form of D(Yoruba, X; Tibetan 1, Tibetan 2). (A) Distribution of D statistics for all pairs of Tibetan samples against five different outgroups. Only when Han Chinese was used as an outgroup, D statistics substantially depart from zero. Grey dotted line marks three standard errors (SE) away from zero. (B) Among Tibetan samples, pairwise differences in longitude strongly correlate with D(Yoruba, Han; Tibetan 1, Tibetan 2). The grey line shows a least square fit and vertical bars represent ± 1 SE.

We first applied several methods to summarize patterns of genetic variation within East Asia, including the Tibetan plateau and Himalaya. Our analyses consistently suggest that Tibetans are genetically structured due to varying level of gene flow with non-Tibetan East Asians. First, principal component analysis (PCA) separated the Tibetan samples from other East Asian samples across PC1 (Fig 1B). Populations living in the eastern slopes of the plateau, such as Naxi, Yi and Tu, are located closest to Tibetans in the PCA plot. The Sherpa lie at the end of PC1 and cluster away from the other Tibetan samples in PC3 (S1 Fig), likely due to strong genetic drift [8]. Second, unsupervised genetic clustering analysis using the program *ADMIXTURE* [18] shows that a majority of ancestry in Tibetans appears to be derived from components most highly represented in Tibetans, while they also have varying level of lowland East Asians ancestry (S2 Fig). Third, to investigate the genetic heterogeneity across Tibetans, we calculated Patterson’s D statistic [19] in the form of D(Yoruba, X; Tibetan 1, Tibetan 2) for all pairs of Tibetan samples; this statistic is expected to equal 0 if the populations follow a model of population divergence without gene flow. In this form, a value of D significantly greater than 0 indicates a greater affinity between Tibetan 2 and the outgroup X, i.e. an excess of shared derived alleles between them, while a significantly negative D value indicates greater affinity between Tibetan 1 and X. When western Eurasian populations were used as an outgroup, the D statistics remained well within three standard error (SE) around zero, suggesting no substantial heterogeneity within Tibetans in reference to them (Fig 2A, S3 Fig). However, when East Asian outgroups were used, the D statistics for many pairs of Tibetans significantly deviated from zero, showing that some Tibetan samples are genetically closer to lowland East Asians than the others (Fig 2A, S3 Fig). Last, we tested for gene flow using the *f*_*3*_ statistic which is negative when the target population can only be modeled as a mixture of groups related to the two reference samples [19]. All Tibetan samples, except for the western most ones in our study (i.e. those from northern Nepal: Sherpa, Tsum and Upper Mustang), showed a negative *f*_*3*_ statistic with another Tibetan and non-Tibetan groups as references (*f*_*3*_ = -5.6 to -0.24 SE; S2 Table), further providing evidence for gene flow between non-Tibetans and one of the two Tibetan populations in the comparison. These results strongly support the idea that there was substantial gene flow between most Tibetan populations and low altitude East Asians; importantly, the difference in D and *f*_*3*_ test results across Tibetans indicates that levels of gene flow varied across these populations, resulting in appreciable genetic heterogeneity.

**Fig 3:**
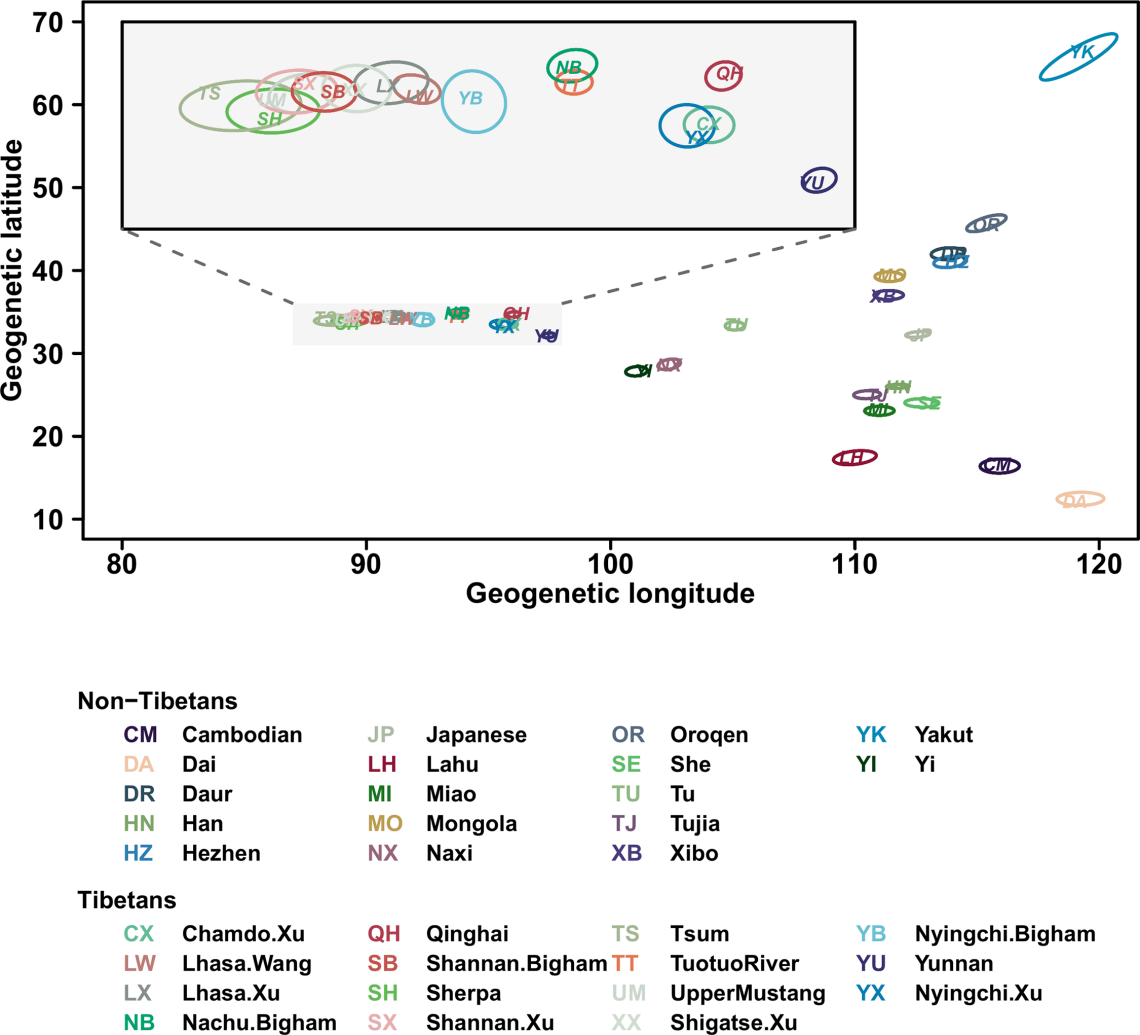
“Geogenetic” locations of East Asian and Tibetan populations inferred from the SpaceMix program. Tibetan samples lined up along geogenetic longitude. The inset shows a zoom in of Tibetans. Grey circles show 95% credible ellipses.

Next, we found that this gene flow occurred mainly along a longitudinal axis creating an East-West genetic cline. Specifically, we found a significant correlation (*r* = 0.73, Mantel test *p* < 0.001; Fig 2B), across pairs of Tibetan samples, between longitudinal distances and differences in genetic affinity with lowland East Asians, measured by Patterson’s D (Yoruba, East Asian; Tibetan 1, Tibetan 2). A leave-one-population-out procedure did not affect the results (*r* = 0.68–0.83, Mantel test *p* < 0.001), confirming that this pattern is not driven by outlier samples. In contrast, there was no such correlation with latitude (Mantel test *p* = 0.48; S4 Fig). We further investigated the direction of the genetic cline and patterns of gene flow in Tibetans by using the SpaceMix program to build a “geogenetic map” of East Asia, in which samples locate on the map reflecting their genetic distance [20]. Assuming a model in which the amount of gene flow between two locations decreases as a function of distance, i.e. isolation by distance, this program provides a two-dimensional representation of sampled populations that reflects their genetic similarity. Specifically, it assumes that genetic covariance between populations decays with distance in a hypothetical two-dimensional plane (“geogenetic space”), and estimates for each population a location which best explains the observed genetic covariance. The inferred geogenetic locations fit the observed genetic covariance between population pairs well, suggesting that the isolation-by-distance model is a reasonable approximation for the sampled populations without requiring additional major long-range migration events (S5 Fig). In the inferred geogenetic space, Tibetan samples lined up along geogenetic longitude (Fig 3), which correlates well with geographic longitude (*r*^*2*^ = 0.76 in Tibetans). Allowing for long-range gene flow in the model did not detect substantial long-range gene flow into any of the Tibetan samples (all below 10%) and found nearly identical geogenetic locations for all populations (*r*^*2*^ ≥ 0.99). Mirroring geography, geogenetic longitudes showed strong correlations with D statistics (*r* = 0.72–0.77, Mantel test *p* < 0.001, S6 Fig).

To obtain an alternative visualization of the spatial pattern of gene flow, we also applied the program EEMS (estimated effective migration surfaces), which infers an “effective migration surface” across a spatial grid from a pairwise genetic distance matrix [21]. As in the SpaceMix results, we find that the isolation-by-distance model in EEMS is sufficient to explain the observed genetic distances, without requiring major long-distance gene flow events (S7 Fig). EEMS detected a clear barrier to gene flow surrounding the Tibetan plateau, which highlights the genetic distinctness of Tibetans as a whole (S8A Fig). Within Tibet, a weak barrier was inferred between Shannan and Nyingchi; denser geographic sampling will be necessary to determine whether this finding truly reflects heterogeneity in the migration rate within Tibet (S8B Fig). In contrast to high connectivity between sites within the plateau, the Tibetan samples from Nepal showed lower migration rates with other samples in the plateau and with each other. These samples also have high levels of linkage disequilibrium (LD), suggesting a role for genetic drift in their differentiation from the rest of Tibetans (S9 Fig).

To summarize, our results clearly show that a longitudinal cline is a major feature of Tibetan population structure and that this cline is likely to be an outcome of gene flow with lowland East Asians, the magnitude of which increases as one moves eastwardly (Fig 2). Our findings are consistent with the previously reported signals of admixture identified in several Tibetan samples using Sherpa and lowland East Asian samples as reference populations [8]. Here, we further expand those conclusions by showing that the inferred admixture follows a pattern of isolation-by-distance along the longitudinal axis.

Previous studies of the genetic history of Tibetans focused on the presence of the Y chromosome haplogroup D, which is shared with Ainu of Japan, but is otherwise absent in East Asia [4]. More recently, Tibetans have been studied extensively for their genetic adaptations to high altitudes [1,2,3]. In this study, we describe the geographic structure of this large ethnic group comprised of millions of individuals occupying a vast territory of rugged terrain by combining most of the published genome-wide variation data of Tibetans with new data from Tibetans in Himalaya. Our findings have several implications. Firstly, they show that, especially on its eastern side, the Tibetan plateau forms a porous barrier to gene flow. Nearby non-Tibetan populations, such as Naxi, Yi and Tu, are genetically closer to Tibetans compared to other lowland East Asians, extending the Tibetan genetic cline to the outskirts of the plateau (S10 Fig). However, the current data do not allow us to infer the direction of historical gene flow in this region. Secondly, the geographic structure of Tibetan populations provides a unique opportunity to investigate how natural selection interacts with gene flow, for example by contrasting the frequency of advantageous alleles over geographic space to those of neutral variants. Unfortunately, the well-known adaptive haplotypes in Tibetans [22,23,24] were not well tagged by the SNPs in our data set, which were limited to the intersection of many different genotyping platforms. With the aid of ancient DNA studies, it may be possible to determine when and where altitude adaptive variants first arose, and how they spread out through time and space in Tibet [25]. Finally, our results help to ameliorate the limited understanding of East Asian population structure and underlying genetic history. Considering the ascertainment bias of genetic markers inherent in all microarray data, we did not try to infer the details of the prehistoric population process leading to the contemporary genetic cline. With more genomic data in the future, it would be of particular interest to investigate the role of the last glacial maximum and of the spread of agriculture in the formation of contemporary population structure in East Asia [26]. Specifically, it is crucial to answer questions such as when the Tibetan gene pool began to diverge from that of lowlanders and when the Tibetan cline began to form. Our study provides a foundation for investigating these questions.

## Materials and methods

### New genotype data

In this study, we used newly generated genome-wide genotype data of 53 unrelated ethnic Tibetan individuals from high altitude regions in the Himalayas, Nepal. Tibetan participants were recruited from two districts during spring and summer of year 2012: 23 individuals are from Tsum region in Gorkha district and 30 individuals are from Upper Mustang region in Mustang district. All participants were born and raised in high altitude regions (≥ 3,000 m). These 53 individuals are a subset of a bigger cohort recruited at the same time, and selected for this study based on harboring negligible level of South Asian ancestry. Saliva samples were collected using OG-500 Oragene saliva collection kits (DNA Genotek, Inc., Ottawa, ON, Canada) and genomic DNA was extracted using PT-L2P reagents (DNA Genotek, Inc., Ottawa, ON, Canada) following manufacturer’s protocol. Genome-wide genotyping experiments were performed at the Genomics facility at the University of Chicago, using both Illumina HumanCore v1-0 (298,931 markers) and HumanOmniExpress-24 v1.0 (716,503 markers) arrays. Illumina GenomeStudio genotyping module was used for calling genotypes from intensity data, using default parameters (GenCall score threshold 0.15) and cluster files provided by the manufacturer. All study participants provided written informed consent. This study was approved by the IRBs at Case Western Reserve University and University of Chicago, by the Oxford Tropical Research Ethics Committee and by the Nepal Health Research Council.

### Compilation of genotype data

We compiled published genome-wide variation data of world-wide populations, focusing on Tibetan samples. Specifically, the following data sets were combined: Human Genome Diversity Panel (HGDP) samples (*n* = 938) genotyped on Illumina 650Y array [16], the 1000 Genomes Project (1KG) phase 3 samples [17] in *IMPUTE2* imputation reference format (n = 2,504; downloaded from https://mathgen.stats.ox.ac.uk/impute/1000GP_Phas3.tgz), and published Tibetan and Sherpa data (*n* = 285), genotyped on various Illumina and Affymetrix genotyping arrays [1,2,8,11,12,13,14]. S2 Table shows a detailed description of Tibetan cohorts used in this study. Genomic positions were lifted over to positions in GRCh37 using liftOver tool downloaded from http://hgdownload.cse.ucsc.edu/admin/exe/linux.x86_64/liftOver. Autosomal biallelic SNPs were used in the analysis, after removing A/T and G/C SNPs for strand ambiguity. For HGDP data, we included SNPs only if all populations have less than 2 (for populations with ≤ 30 samples) or 3 (for populations with > 30 samples) missing genotypes. For Tibetan data, we removed individuals and SNPs with > 5% missing genotypes and SNPs with Hardy-Weinberg *p*-value < 0.000001 for each study. We randomly removed one individual from three pairs of genetic relatives in Tibetans closer than first cousins. Data filtering and relatedness estimation were performed using PLINK v1.90b3j [27]. Our final genotype data set included 3,780 individuals and 69,427 SNPs.

### Population genetic analysis of the Tibetan genetic cline

Principal component analysis (PCA) was performed using the smartpca program version 10210 in EIGENSOFT 5.0.1 package [28]. 62,691 SNPs with minor allele frequency (maf) ≥ 0.05 in East Asian and Tibetan populations (Fig 1B) were used for PCA. For unsupervised genetic clustering, we used *ADMIXTURE* v1.22 [18]. For this analysis, we used 706 individuals including all East Asians and Tibetans, as well as individuals from HGDP French, Pathan, Papuan, Pima, Karitiana and Uygur and randomly chosen 25 individuals from 1KG ITU (Indian Telugu from the UK) to represent genetic diversity across Eurasia and America. 44,826 SNPs were included in the analysis after pruning for LD with *r*^*2*^ threshold of 0.2. For the numbers of clusters (K) from 2 to 11, we ran 50 replicates with different random seeds and chose one for each K with the highest log likelihoods (LL) as the best run. For all K values, top 10 runs have similar LL values within difference of 1, showing convergence to global optimum. K = 9 was chosen as the best K values based on the smallest value of five-fold cross validation error. Three Tibetan samples (one from each of Nyingchi.Xu, Lhasa.Xu and TuotuoRiver cohorts) showed genetic affinity with lowland East Asians much higher than the rest of their cohorts and therefore excluded from further population-based analyses. One Papuan and two Yi individuals from HGDP were also excluded as outliers.

### Population genetic analysis of geographic structure of the Tibetan genetic cline

Patterson’s D and *f*_*3*_ statistics were calculated for all population sets using *qpDstat* and *qp3Pop* programs in the ADMIXtools v2 package [19]. Standard errors were estimated using 5 cM block jackknifing. Correlations between geographic or geogenetic distances and genetic affinity with lowland East Asians, measured by D(Yoruba, Han; Tibetan 1, Tibetan 2), were tested using Mantel test to take non-independence of pairwise data into account, as implemented in R function “mantel.rtest” in the “Ade4” R package [29]. The SpaceMix program [20] was used to investigate isolation-by-distance pattern of decay of genetic covariance in East Asia and Tibet. SpaceMix estimates population locations in a hypothetical two-dimensional “geogenetic” space which best explain the observed pattern decay of genetic covariance against distance. An additional long distance migration edge can be inferred for each population to model long-range gene flow in addition to local gene flow. For each model, we ran five fast runs for 10^6^ generations and a long run of 10^7^ generations was followed using estimates from the last generation of the best fast run as initial values. A sample was taken in every 10^4^ generations, resulting in 1,000 samples for estimating posterior distribution of each parameter. For models inferring geogenetic location of populations, geographic location was used as a prior. We also applied the EEMS program [21] to our data set for visualizing barriers and corridors of gene flow. Specifically, this method estimates a map of relative effective migration rates and a paired map of effective local diversity rates. This method works by approximating the continuous habitat by a dense grid of subpopulations, and then estimating symmetric nearest-neighbor migration rates (denoted *m* below) and local diversity (denoted *q* below, roughly corresponds to a local *N*_*e*_). In effect, this is done by comparing the expected distances induced by the migration rates with an observed genetic distance matrix, and a posterior distribution is inferred using Markov Chain Monte Carlo (MCMC). For all analyses, we performed 10 replicate runs, each of which consisted of a burn-in of 1,000,000 iterations, and recorded 250 iterations each at a thinning proportion of 0.1%. We then collated the samples from all MCMC-chains and produced contour plots of the posterior means of each parameter over space.

## Supporting information

S1 Fig PCA of East Asian and Tibetan individuals. PC1 and PC3 are plotted. PC3 shows divergence of Sherpa individuals away from the rest of Tibetans, due to strong genetic drift they experienced. Colored circles mark mean positions of populations. Numbers in parenthesis represent proportion of total variation explained by each PC.
(TIF)

S2 Fig *ADMIXTURE* analysis of East Asian populations in conjunction with other non-African populations (K = 2 to 11). Five-fold cross validation was lowest at K = 9. At this K value, Tibetan individuals harbor distinct components as their major ancestry, i.e. orange in Sherpa and green in other Tibetans. However, variable proportions of their ancestry are from components most concentrated in non-Tibetan East Asians, such as navy and blue ones, which are most concentrated in Dai and Yakut, respectively.
(TIFF)

S3 Fig Distribution of Patterson’s D(Yoruba, X; Tibetan 1, Tibetan 2) for all pairs of Tibetan cohorts, using each of 36 Eurasian populations as an outgroup X. No substantial deviation from zero was observed when populations with no East Asian ancestry were used as an outgroup, strongly supporting Tibetan cladeness against them. In contrast, D statistics significantly deviated from zero when any of East Asian populations were used as an outgroup.
(TIF)

S4 Fig A scatter plot between geographic distance in latitude and D(Yoruba, Han; Tibetan 1, Tibetan 2) for all pairs of Tibetan cohorts. In contrast to longitude, there was no correlation between latitudinal distance and genetic affinity with lowland East Asians. The grey line shows a least square fit and vertical bars represent ± 1 SE.
(TIF)

S5 Fig Comparison of isolation-by-distance pattern of decay in genetic covariance, either observed (black dots) or estimated from the SpaceMix program (grey dots). (A) A SpaceMix model using fixed geographic locations does not explain the observed pattern of genetic covariance. (B) A SpaceMix model with inferred “geogenetic” location well fits the observed pattern of genetic covariance decay.
(TIF)

S6 Fig A scatter plot between “geogenetic” distances and D(Yoruba, Han; Tibetan 1, Tibetan 2) for all pairs of Tibetan cohorts. Similar to geographic longitude, geogenetic longitude was strongly correlated with genetic affinity with lowland East Asians. Geogenetic latitude was also correlated with D statistics, but a cohort from southeastern margin of the plateau (“Yunnan”) mainly drove this signal. Pairs including Yunnan Tibetan are marked with grey square dot. The grey line shows a least square fit and vertical bars represent ± 1 SE.
(TIF)

S7 Fig A comparison of observed genetic dissimilarity (y-axis) with estimates from the EEMS program (x-axis). (A) All East Asian and Tibetan samples and (B) all Tibetan samples and Naxi, Yi and Tu. The model estimates show a good fit with the observed data.
(TIFF)

S8 Fig An “Effective migration surface” inferred across the Tibetan plateau and surrounding region using the EEMS program. (A) All East Asian and Tibetan samples and (B) all Tibetan samples and Naxi, Yi and Tu. Brown and blue colors represent areas of low and high gene flow, respectively. Barriers to gene flow were estimated around the Himalayas and, to a lesser extent, between central and eastern Tibet. Lhasa, the capitol of Tibet Autonomous Region, shows an increased connection in comparison to the surrounding area.
(TIFF)

S9 Fig Decay of LD measured by average r^2^ between diploid genotypes of markers against genetic distance in centiMorgan (cM) scale. (A) 32 East Asian cohorts with minimum sample size of 8. (B) 12 East Asian cohorts with minimum sample size of 19. For each, we randomly sampled the corresponding number of samples (either 8 or 19) to match sample size across all populations. Tibetan cohorts from Nepal (Sherpa, Tsum and UpperMustang), together with Yakut from southern Siberia, show elevated LD, reflecting strong genetic drift they experienced.
(TIF)

S10 Fig Correlations of geographic distances and genetic affinity with Han Chinese, measured by Patterson’s D(Yoruba, Han; Pop 1, Pop 2). Pairwise distance and D statistic were calculated between all pairs Tibetan cohorts and nearby populations (Naxi, Yi and Tu). (A) A correlation between longitudinal distance and D statistic was well maintained after including three non-Tibetan populations. (B) No latitudinal correlation was found even after including three non-Tibetan populations. Vertical bars represent ± 1 SE. Grey dots represent pairs including non-Tibetan populations, with squares, triangles and circles representing Naxi, Yi and Tu, respectively.
(TIF)

S1 Table Tibetan cohorts analyzed in this study (PDF)

S2 Table The most negative *f*_*3*_ statistic for each Tibetan cohort, with at least one non-Tibetan population was included in the reference pair. (PDF)
