# Correction: A longitudinal cline characterizes the genetic structure of human populations in the Tibetan plateau

**DOI:** 10.1371/journal.pone.0183407

**Published:** 2017-08-10

**Authors:** Choongwon Jeong, Benjamin M. Peter, Buddha Basnyat, Maniraj Neupane, Cynthia M. Beall, Geoff Childs, Sienna R. Craig, John Novembre, Anna Di Rienzo

The following information is missing from the Funding section: This work was supported in part by National Science Foundation grant 1153911 to CMB.

## References

[1] Jeong C, Peter BM, Basnyat B, Neupane M, Beall CM, Childs G, et al (2017) A longitudinal cline characterizes the genetic structure of human populations in the Tibetan plateau. PLoS ONE 12(4): e0175885 https://doi.org/10.1371/journal.pone.0175885 28448508 10.1371/journal.pone.0175885 PMC5407838

