# Supplementary figures and images for: A longitudinal cline characterizes the genetic structure of human populations in the Tibetan plateau
Source: PLoS One. 2017 Apr 27;12(4):e0175885. doi: 10.1371/journal.pone.0175885 (PMC5407838; doi:10.1371/journal.pone.0175885)

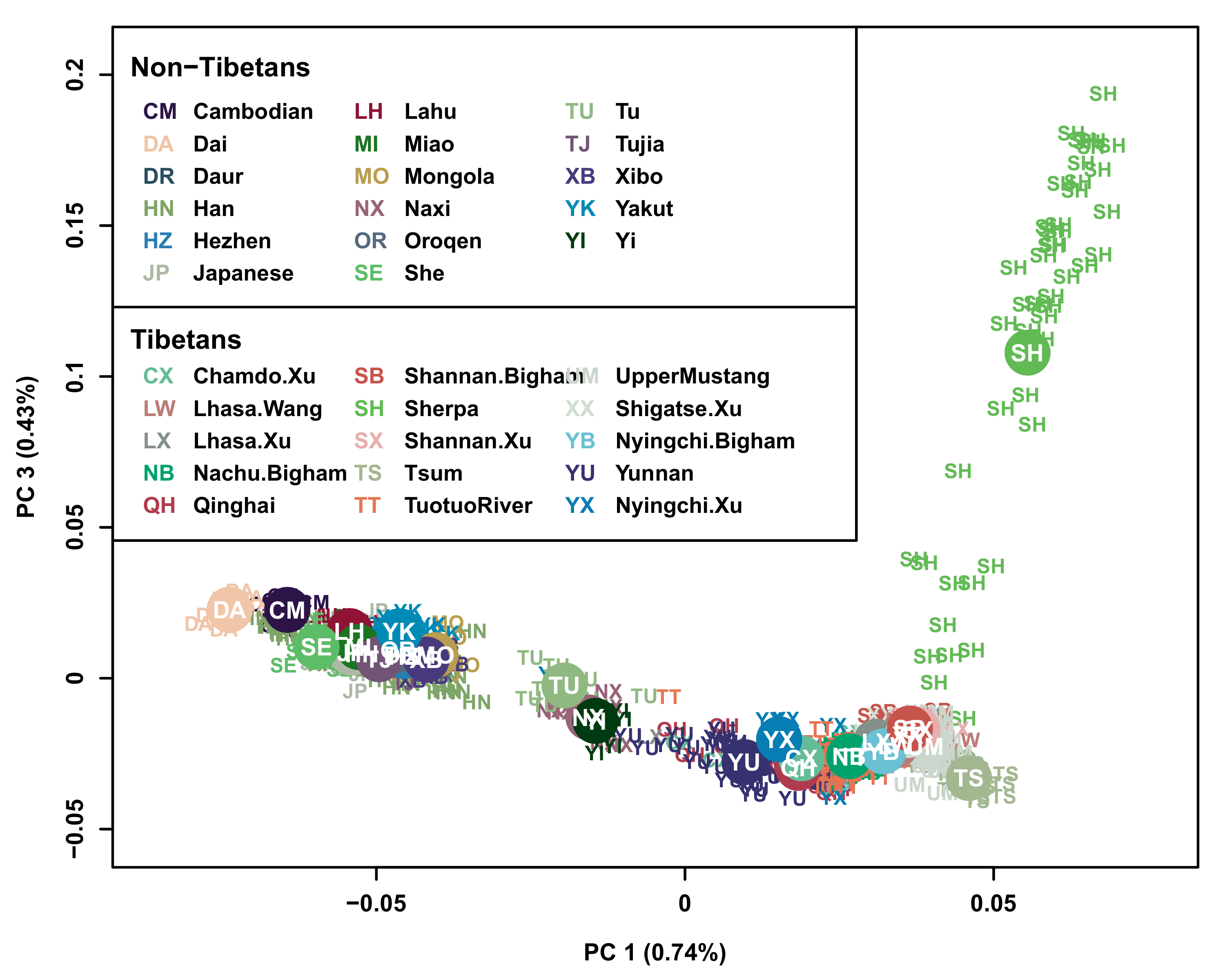

Supplement: S1 Fig — PC1 and PC3 are plotted. PC3 shows divergence of Sherpa individuals away from the rest of Tibetans, due to strong genetic drift they experienced. Colored circles mark mean positions of populations. Numbers in parenthesis represent proportion of total variation explained by each PC. (TIF) [file pone.0175885.s001.tif]

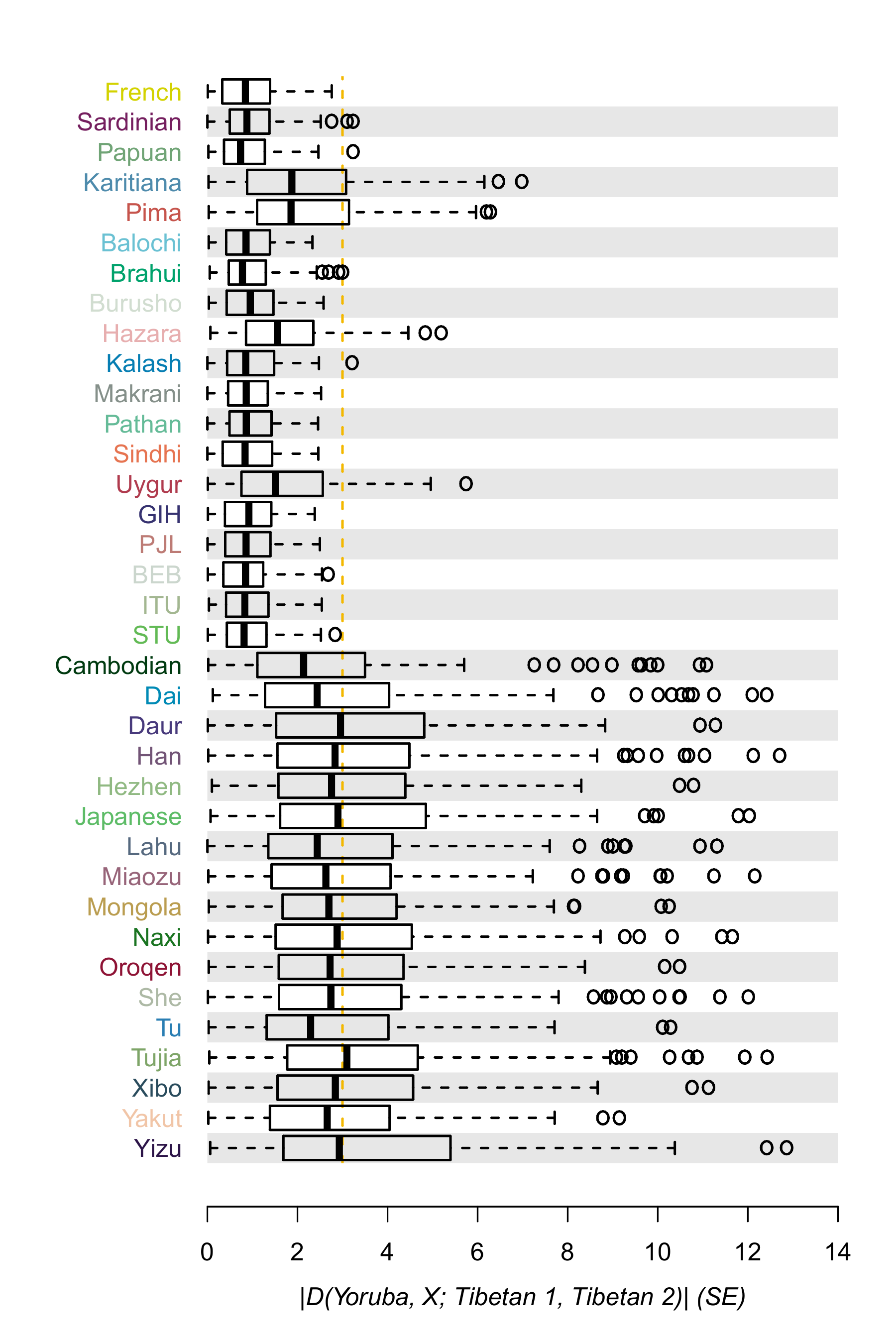

Supplement: S3 Fig — No substantial deviation from zero was observed when populations with no East Asian ancestry were used as an outgroup, strongly supporting Tibetan cladeness against them. In contrast, D statistics significantly deviated from zero when any of East Asian populations were used as an outgroup. (TIF) [file pone.0175885.s003.tif]

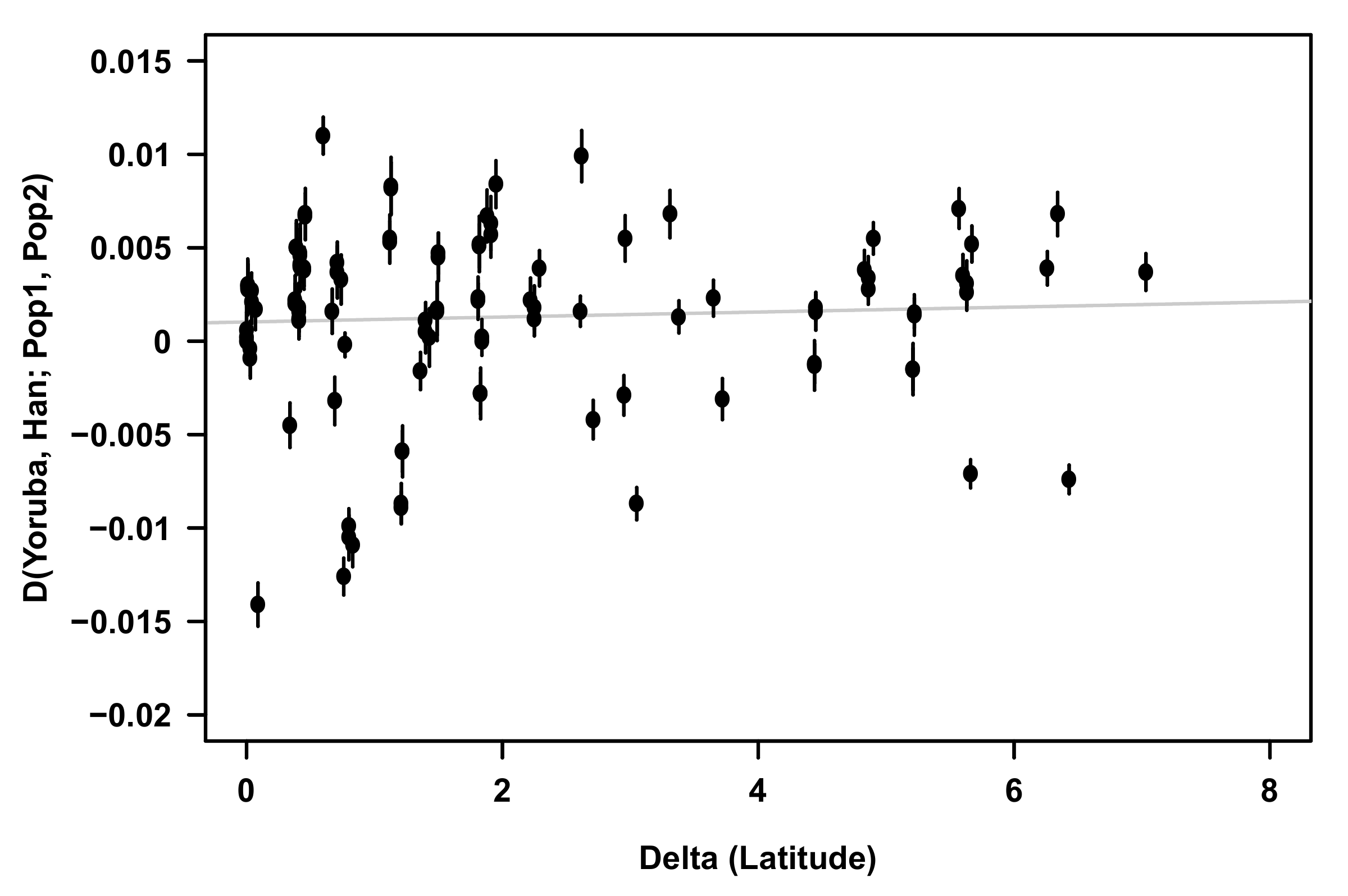

Supplement: S4 Fig — In contrast to longitude, there was no correlation between latitudinal distance and genetic affinity with lowland East Asians. The grey line shows a least square fit and vertical bars represent ± 1 SE. (TIF) [file pone.0175885.s004.tif]

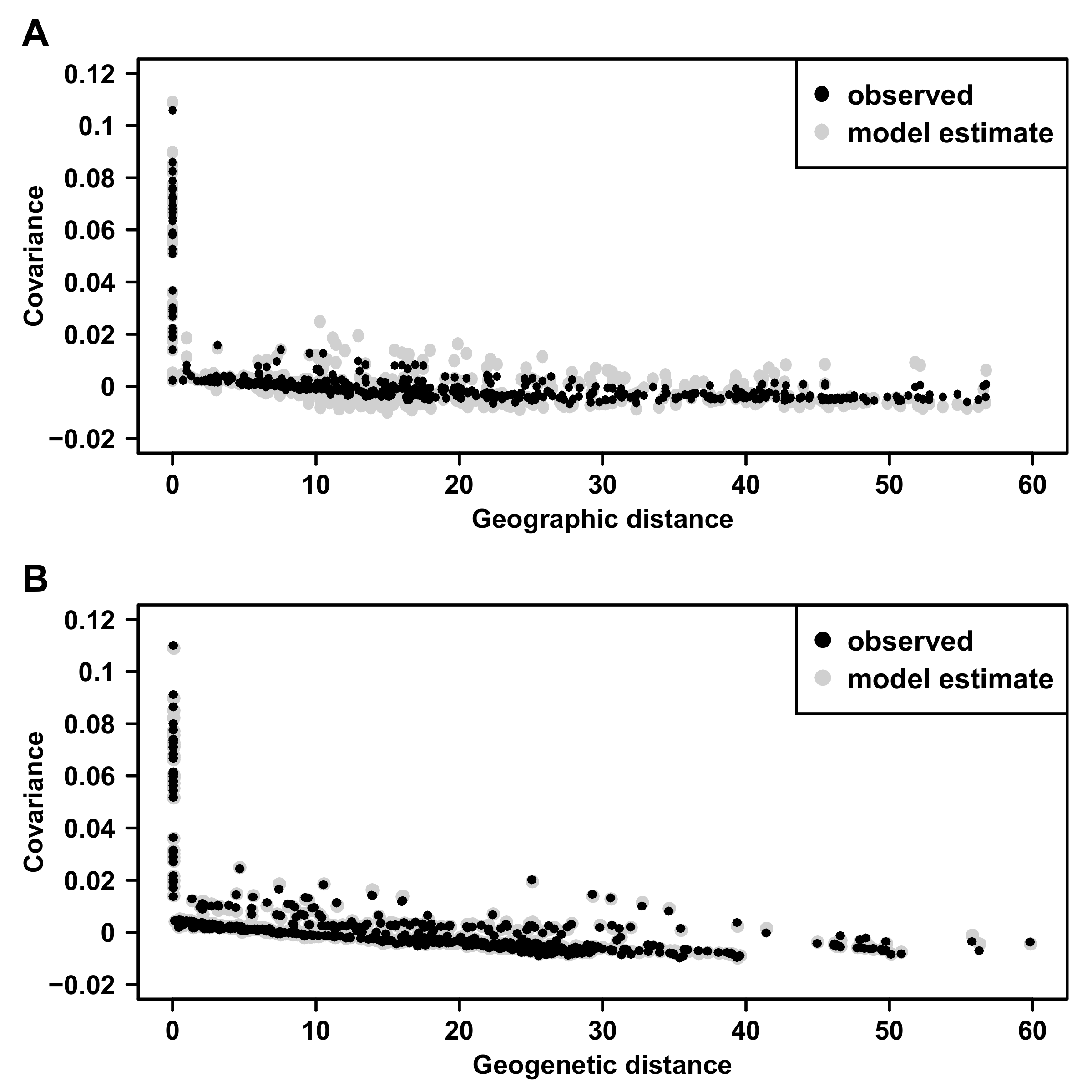

Supplement: S5 Fig — (A) A SpaceMix model using fixed geographic locations does not explain the observed pattern of genetic covariance. (B) A SpaceMix model with inferred “geogenetic” location well fits the observed pattern of genetic covariance decay. (TIF) [file pone.0175885.s005.tif]

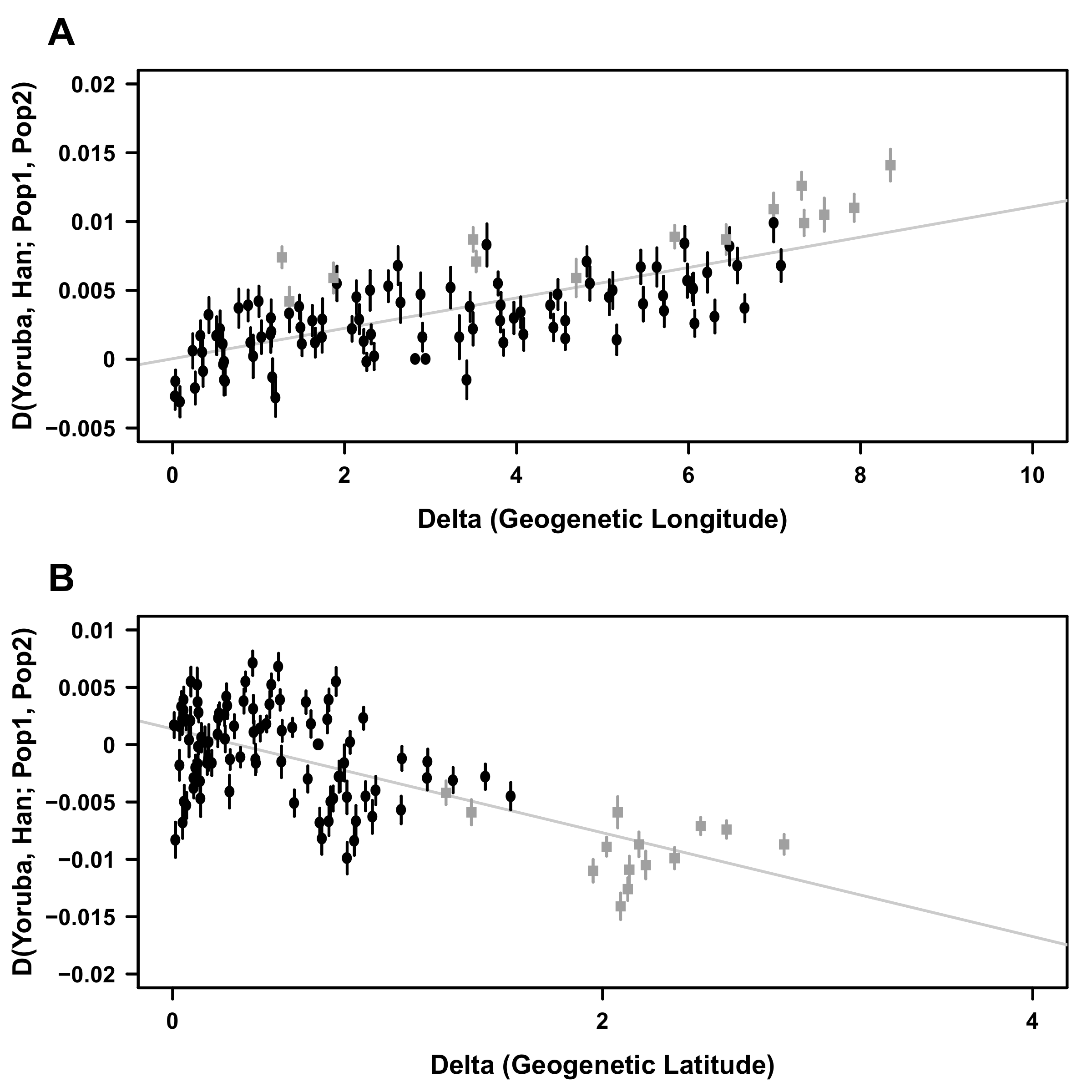

Supplement: S6 Fig — Similar to geographic longitude, geogenetic longitude was strongly correlated with genetic affinity with lowland East Asians. Geogenetic latitude was also correlated with D statistics, but a cohort from southeastern margin of the plateau (“Yunnan”) mainly drove this signal. Pairs including Yunnan Tibetan are marked with grey square dot. The grey line shows a least square fit and vertical bars represent ± 1 SE. (TIF) [file pone.0175885.s006.tif]

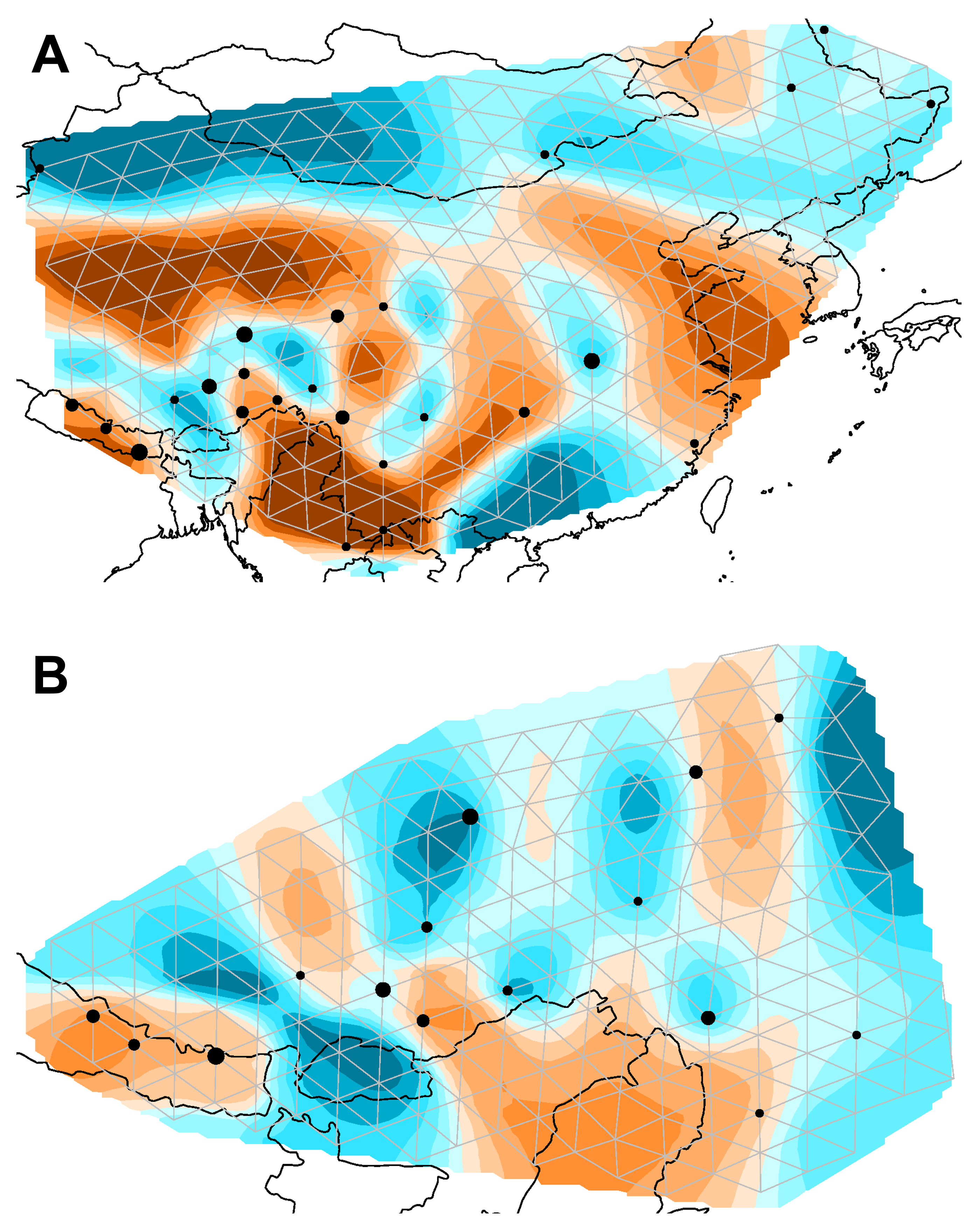

Supplement: S8 Fig — (A) All East Asian and Tibetan samples and (B) all Tibetan samples and Naxi, Yi and Tu. Brown and blue colors represent areas of low and high gene flow, respectively. Barriers to gene flow were estimated around the Himalayas and, to a lesser extent, between central and eastern Tibet. Lhasa, the capitol of Tibet Autonomous Region, shows an increased connection in comparison to the surrounding area. (TIFF) [file pone.0175885.s008.tiff]

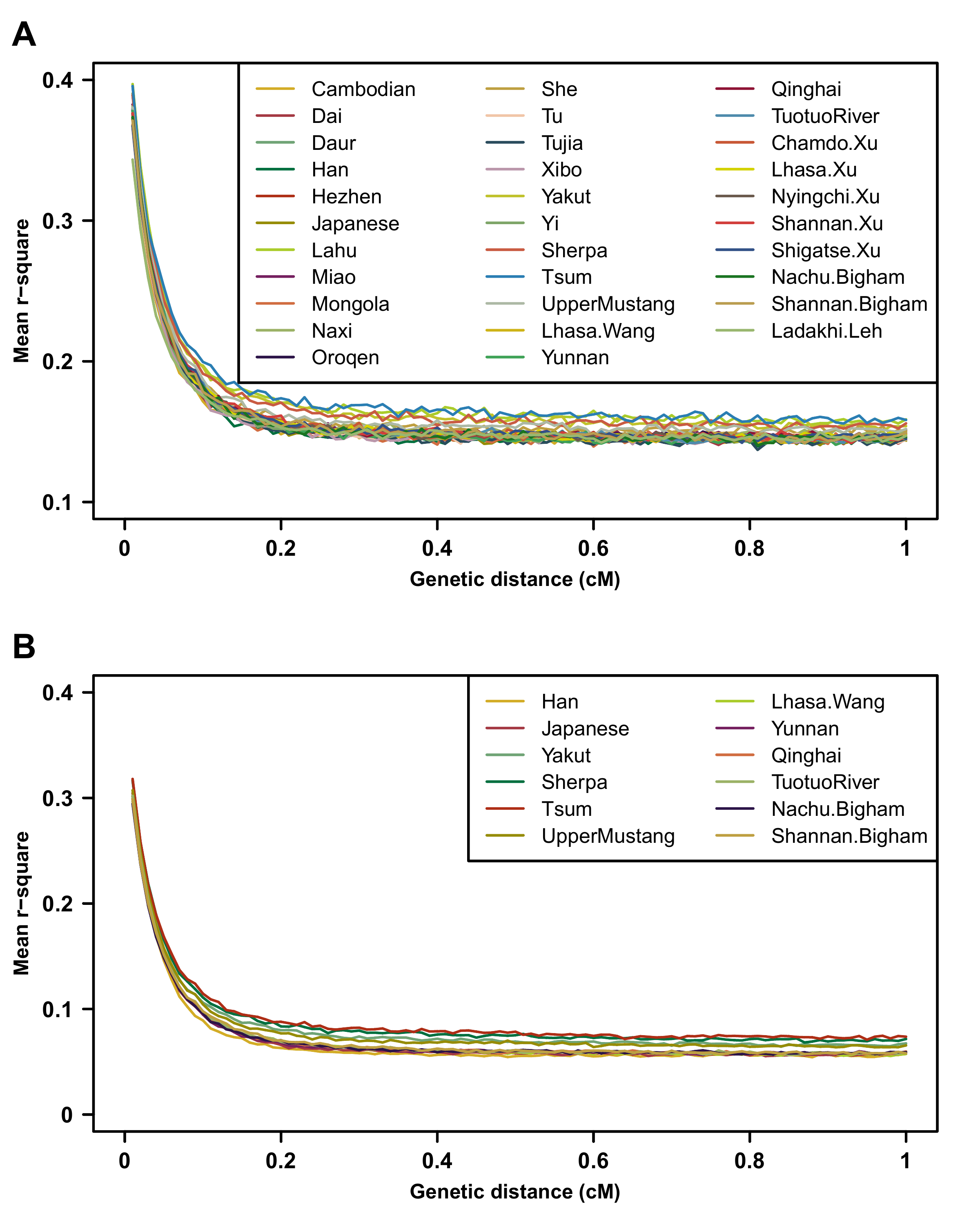

Supplement: S9 Fig — (A) 32 East Asian cohorts with minimum sample size of 8. (B) 12 East Asian cohorts with minimum sample size of 19. For each, we randomly sampled the corresponding number of samples (either 8 or 19) to match sample size across all populations. Tibetan cohorts from Nepal (Sherpa, Tsum and UpperMustang), together with Yakut from southern Siberia, show elevated LD, reflecting strong genetic drift they experienced. (TIF) [file pone.0175885.s009.tif]

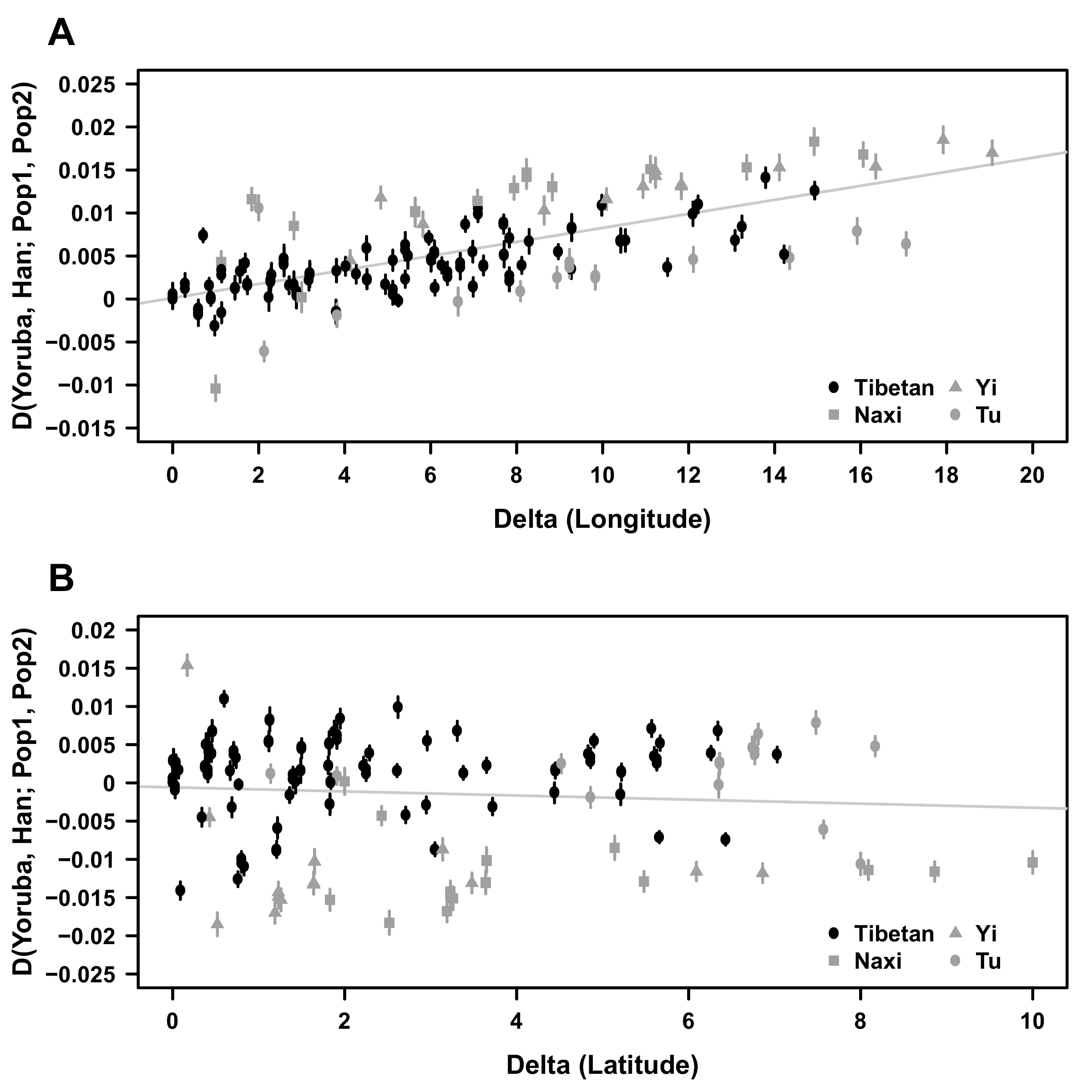

Supplement: S10 Fig — Pairwise distance and D statistic were calculated between all pairs Tibetan cohorts and nearby populations (Naxi, Yi and Tu). (A) A correlation between longitudinal distance and D statistic was well maintained after including three non-Tibetan populations. (B) No latitudinal correlation was found even after including three non-Tibetan populations. Vertical bars represent ± 1 SE. Grey dots represent pairs including non-Tibetan populations, with squares, triangles and circles representing Naxi, Yi and Tu, respectively. (TIF) [file pone.0175885.s010.tif]
