# Supplementary material for: A longitudinal cline characterizes the genetic structure of human populations in the Tibetan plateau
Source: PLoS One. 2017 Apr 27;12(4):e0175885. doi: 10.1371/journal.pone.0175885 (PMC5407838; doi:10.1371/journal.pone.0175885)
Supplement: S1 Table — (PDF) [file pone.0175885.s011.pdf]

S1 Table. Tibetan cohorts analyzed in this study

Cohort	Location[1]	Lat[2]	Long[3]	N	Platform[4]	Study
Sherpa	Khumbu, Nepal	27.83	86.65	49	Omni1-Quad	Jeong et al 2014
Tsum	Gorkha, Nepal	28.52	85.08	23	OmniExpress / HumanCore+	This study
Upper.Mustang	Mustang, Nepal	29.19	83.94	30	OmniExpress / HumanCore+	This study
Lhasa.Wang	Lhasa, TAR[5]	29.64	91.17	30	Human1M-Duo	Wang et al 2011
Yunnan	Yunnan	28.43	98.87	35	Human610-Quad	Beall et al 2010
Qinghai	Madoi, Qinghai	34.86	98.16	31	Affymetrix 6.0	Simonson et al 2010
TuotuoRiver	Tuotuo, Qinghai	34.09	92.91	46	Affymetrix 6.0	Wuren et al 2014
Chamdo.Xu	Chamdo, TAR	31.14	97.18	9	Affymetrix 6.0	Xu et al 2011
Lhasa.Xu	Lhasa, TAR	29.64	91.17	11	Affymetrix 6.0	
Nyingchi.Xu	Nyingchi, TAR	29.65	94.36	10	Affymetrix 6.0	
Shannan.Xu	Shannan, TAR	29.23	91.77	9	Affymetrix 6.0	
Shigatse.Xu	Shigatse, TAR	29.26	88.89	9	Affymetrix 6.0	
Nachu.Bigham	Nachu, TAR	31.48	92.06	21	Affymetrix 6.0	Bigham et al 2010
Nyingchi.Bigham	Nyingchi, TAR	29.65	94.36	6	Affymetrix 6.0	
Shannan.Bigham	Shannan, TAR	29.23	91.77	19	Affymetrix 6.0	

[1] Sampling location in China, except for cohorts specified as "Nepal"

[2,3] Latitude (north) and Longitude (east) of sampling locations, obtained from Google Map if not specified in the original study

[4] Names of Illumina genotyping array (except for Affymetrix 6.0) used for data generation

[5] TAR = Tibet Autonomous Region in China
