# Supplementary material for: A longitudinal cline characterizes the genetic structure of human populations in the Tibetan plateau
Source: PLoS One. 2017 Apr 27;12(4):e0175885. doi: 10.1371/journal.pone.0175885 (PMC5407838; doi:10.1371/journal.pone.0175885)
Supplement: S2 Table — (PDF) [file pone.0175885.s012.pdf]

S2 Table. The most negative f_3 statistic for each Tibetan cohort, with at least one non-Tibetan population was included in the reference pair.

Target	Ref 1	Ref 2	Z
Lhasa.Wang	Tujia	Shannan.Xu	-3.330
Yunnan	Dai	TuotuoRiver	-4.519
Qinghai	Kalash	Yunnan	-4.786
TuotuoRiver	Kalash	Yunnan	-3.449
Chamdo.Xu	Tujia	Lhasa.Xu	-3.295
Lhasa.Xu	Naxi	Shannan.B	-3.302
Nyingchi.Xu	Miao	Snannan.Xu	-4.022
Shannan.Xu	Daur	Tsum	-1.341
Shigatse.Xu	BEB	Shannan.Xu	-1.631
Nachu.Bigham	Kalash	Yunnan	-4.566
Nyingchi.Bigham	Oroqen	Tsum	-0.237
Shannan.Bigham	BEB	Shannan.Xu	-1.730
